# Learning Theory-Driven Tips for Designing Effective Learning Solutions for the Continuous Education of Community Pharmacists to Enhance Patient-Centered Care—A Qualitative Study

**DOI:** 10.3390/healthcare10071167

**Published:** 2022-06-22

**Authors:** Magdalena Cerbin-Koczorowska, Piotr Przymuszała, Sandra Fabianowska, Natalia Gałązka, Łucja Zielińska-Tomczak

**Affiliations:** 1 Department of Medical Education, Poznan University of Medical Sciences, 7 Rokietnicka St., 60-806 Poznan, Poland; pprzymuszala@ump.edu.pl (P.P.); zielinskatomczak@ump.edu.pl (Ł.Z.-T.); 2 Students’ Scientific Club of Medical Education, Department of Medical Education, Poznan University of Medical Sciences, 7 Rokietnicka St., 60-806 Poznan, Poland; sandrafabianowska96@gmail.com (S.F.); s83002@student.ump.edu.pl (N.G.)

**Keywords:** continuous education, lifelong learning, pharmacists, andragogy, pharmaceutical care

## Abstract

The constant development of medical and pharmaceutical sciences and the changing roles of pharmacists highlight the importance of lifelong learning in their profession. Given the identified knowledge gaps in the literature in terms of pharmacists’ preferences for lifelong learning, the study aimed to evaluate the opinions and attitudes of community pharmacists towards lifelong learning, including their previous experiences and educational needs, in order to propose evidence-based tips for designing such solutions and interventions intended for them both in face-to-face and online forms. For this purpose, ten semi-structured in-depth interviews were conducted with Polish community pharmacists on the topic using a thematic guide. Subsequently, they were subjected to literal transcription and interpretative phenomenological analysis by two independent researchers using phenomenology as the qualitative approach. The identified themes covered the topic’s relevance for pharmacists’ work, practice-oriented form and content, previous learners’ experiences as a foundation for further learning, commercial initiatives’ risks, motivation sources, and barriers for participation in lifelong learning solutions so far. Based on the insights provided by the respondents, as well as scientifically proven learning theories and educational principles, ten tips were formulated for designing recipient-friendly learning solutions and interventions within the framework of postgraduate lifelong learning of pharmacists.

## 1. Introduction

World Health Organization (WHO) experts describing the professional roles of pharmacists pay much attention to education [1]. Importantly, the seven most important aspects of the profession include, among others, the readiness to share the possessed expert knowledge with patients and other healthcare professionals. Although, until recently, the scope of this expertise was drug-oriented—its preparation and durability preservation, the implementation of a wide range of pharmaceutical services realized in subsequent countries based on pharmaceutical care-based philosophy has focused the activities of pharmacists more on the patient. In view of the growing demand for services in the healthcare sector, pharmacists are involved, for instance, in designing patient pharmacotherapy, caring for treatment optimization, and other activities, building their position as full members of the medical team [2,3,4]. Moreover, since the beginning of the COVID-19 pandemic, which has severely impaired and overburdened the functioning of healthcare systems in many countries, policy and decision makers are becoming more and more willing to extend the scope of their professional activities [5,6].

In Poland, this extension included, inter alia, broadening the scope of pharmacists’ competencies in issuing prescriptions and performing vaccinations [5]. In addition, the Act on the Pharmacist’s Profession of 10 December 2020 [7] recently extended the pharmacists’ scope of practice with a number of new competencies, such as performing a drug review, issuing a repeat prescription, or developing an individual pharmaceutical care plan. However, despite the positive attitudes of the majority of this professional community, the simultaneous skepticism resulting from the low sense of competence needed to perform new duties cannot be ignored and may stand in the way of the dissemination of the services described in the Act [8]. The need to implement appropriate educational solutions has been identified by experts at the Ministry of Health, who, in their report, considered courses broadening the qualifications of the personnel as a necessary condition for the general implementation of the extensions in the scope of practice assumed in the Act on the Pharmacist’s Profession [9].

The changing role of the pharmacist in the healthcare system increases the importance of the readiness to participate in lifelong learning, as described by WHO [1]. Pursuant to the Act of 6 September 2001, Pharmaceutical Law [10], “a pharmacist employed in a pharmacy (...) is obliged to improve their professional qualifications by participating in continuous training to update their knowledge and constantly educate themselves”. Meanwhile, the current pandemic involves, on the one hand, an increase in the scope of pharmacists’ competencies, and on the other hand, the process of acquiring new knowledge may be impeded due to a significant work-overload [11]. Simultaneously, other authors notice that there is a need not only for continuing education of pharmacists but also for ensuring the educational value and effectiveness of continuous education [12].

The systematic review by Micallef and Kayyali [13] showed a knowledge gap in terms of pharmacists’ preferences for lifelong learning. Meanwhile, satisfaction from participation in the educational intervention, described as the first level of the revised Kirkpatrick Model [14], is fundamental to the willingness to engage in the learning process and, in consequence, to achieve the assumed learning outcomes. Therefore, the aim of this study was to evaluate the opinions and attitudes of community pharmacists towards lifelong learning, their previous experiences with its different forms, and their educational needs in an attempt to increase their satisfaction and willingness to participate in lifelong learning initiatives. Hence, an additional aim of the study was to formulate evidence-based tips for designing lifelong learning solutions and interventions for the continuous development of community pharmacists. Given the ongoing COVID-19 pandemic, the growing popularity of online education, and the specificity of e-learning, the proposed tips were also prepared to cover online solutions with an emphasis on new possibilities arising from their use or any potential differences in comparison with face-to-face teaching.

## 2. Materials and Methods

### 2.1. Researchers’ Characteristics

The principal researcher is a pharmacist with a PhD degree and MSc in Clinical Education, experienced in qualitative research methods. Researchers with other backgrounds and experiences were involved in the study to minimize the risk of potential bias. The second researcher is a physician with an experience in qualitative methodology. The third author is a recent pharmacy graduate, and the fourth is a pharmacy student, which allows them to interpret the data from a different perspective due to no experience participating in postgraduate lifelong learning. The last author is also a pharmacist with an additional Bachelor’s degree in Public Health, an additional asset during data analysis. Apart from the second researcher (P.P.) who is a male, all other researchers were females.

### 2.2. Study Design

In order to obtain richer and more insightful data on community pharmacists’ opinions, attitudes, and points of view towards lifelong learning than it would be possible to obtain if we used quantitative methods, the decision was made to collect data using qualitative methodology [15,16]. It not only produces more detailed data but also allows for the ‘understanding’ of the phenomenon instead of proving the previously stated hypothesis [17].

We used the phenomenological approach to explore the personal experiences, perspectives, and motivations of respondents [18,19]. The literature describes two types of phenomenology. The first is descriptive (Husserlian), assumes that the researcher should achieve the state of ‘presuppositionlessness’ and launch the study as a tabula rasa to identify the ‘objective reality’. In contrast, the interpretive approach (Heideggerian) states that it is impossible to eliminate the researcher bias, and that the interaction between the researcher and the subject (‘intersubjectivity’) allows for a more accurate interpretation of the described experiences [20].

Given the educational backgrounds and a personal interest of the main researcher in the subject of continuous education of pharmacists, the interpretive phenomenological approach (IPA) was considered more appropriate. Brochures with invitations to participate in the study were sent to the managers of pharmacies by e-mail with requests to pass it on to their employees and included information about the aims of the project, and its scientific, noncommercial, and voluntary character. The inclusion criteria in the sample included being a professionally active pharmacist working in the community pharmacy and the consent to participate in the study. Convenience sampling was used. We did not introduce any restrictions for potential participants in regard to their age, gender, or specialization, but we collected relevant demographic information on it from the respondents, which enabled differentiating within the sample. Since the information on the study was provided to the managers of pharmacies, we do not have information on dropouts or people refusing to participate—only those interested in participating contacted the investigators. Consequently, ten face-to-face semi-structured interviews were conducted in May 2017 with pharmacists working in community pharmacies in Poznan (Poland). The duration time of the interviews ranged between 19 min 24 s and 39 min 19 s (28 min 2 s on average). Demographic data on the respondents are provided in Table 1.

The interviews were carried out using a thematic guide and covered a list of topics and questions presented in Table 2. The interviews were conducted during the respondents’ working hours but outside of their workplace to ensure their freedom of expression. For participants’ convenience we chose a neutral place close to their workplace, for instance a coffeehouse or a park. No one else was present during the interviews besides the researcher and the participant.

### 2.3. Data Processing and Analysis

The principal researcher (M.C.-K.) conducted all interviews. Given her educational and professional backgrounds as a pharmacist rendering it impossible for her to definitively give up her own beliefs about participation in continuous education, to ensure as high data validity as possible, the ‘fore-structure’ concept of IPA was used. Both at the stage of data collection and its subsequent analysis, the researcher tried to present the attitude of reflexivity and self-scrutiny [21,22]. Interviewer and participants did not know each other before the study. Therefore, at the beginning of each interview, the principal investigator introduced herself. She also indicated that she was a pharmacist, gave information on her place of employment, and explained that the study was conducted at a medical university as the first stage of a larger project, and that its result would allow for better design of further quantitative research projects. The study protocol assumed a one-time meeting between the researcher and the respondent. To maintain high-quality of data collection, the interviews were recorded using a Sony ICDTX50 audio recorder. Each session was preceded by obtaining consent for recording, discussing the study protocol, and clarifying any doubts. During the interviews, no data allowing respondents’ identification were collected. The basis for the data analysis were the transcripts of the recordings. During the interview, in order not to interrupt the participant, the interviewer was making notes only on the topics that appeared in the respondents’ statements, to which she wanted to come back later. After the data collection process, the recordings were encoded and transcribed. Transcripts were then subjected to interpretative phenomenological analysis with the use of ATLAS.ti software by two researchers (researcher’s triangulation) to broaden the research perspective and confirm the research results [23]. It followed the stages described by Pietkiewicz and Smith [24]: repeated reading and notes taking, then transforming these notes into emerging themes, finding relationships, and finally grouping the themes. The interpretative phenomenological analysis was implemented during the data analysis process in order to better understand respondents’ experiences [25]. The two aforementioned researchers started the analysis of data by independently reading the transcripts multiple times and taking notes. Next, they used these notes to form emerging themes and find relationships between them. Then they compared and discussed their results (emerging themes and relationships between them) to group the themes and produce the final report. An example of the data analysis process was presented as the Supplementary File S1. Data saturation was also debated by the researchers. The team concluded that the obtained data allowed to answer the research question posed as the study aim. It was also decided that further data collection may take place based on a questionnaire, which is planned to be the subject of further research.

### 2.4. Ethical Issues

The study’s project was presented to the Bioethical Committee of the Poznan University of Medical Sciences, which confirmed that its approval was not necessary according to Polish law [26]. Nevertheless, we paid attention during the study to adhere to the highest ethical standards. Potential respondents were notified about the aims, methods of data collection used in the study, as well as its anonymous and voluntary character prior to starting the interviews. At this stage, informed consent was also obtained from them, and they were made aware that at any stage of the study, they could resign from further participation.

## 3. Results

**Figure 1:**
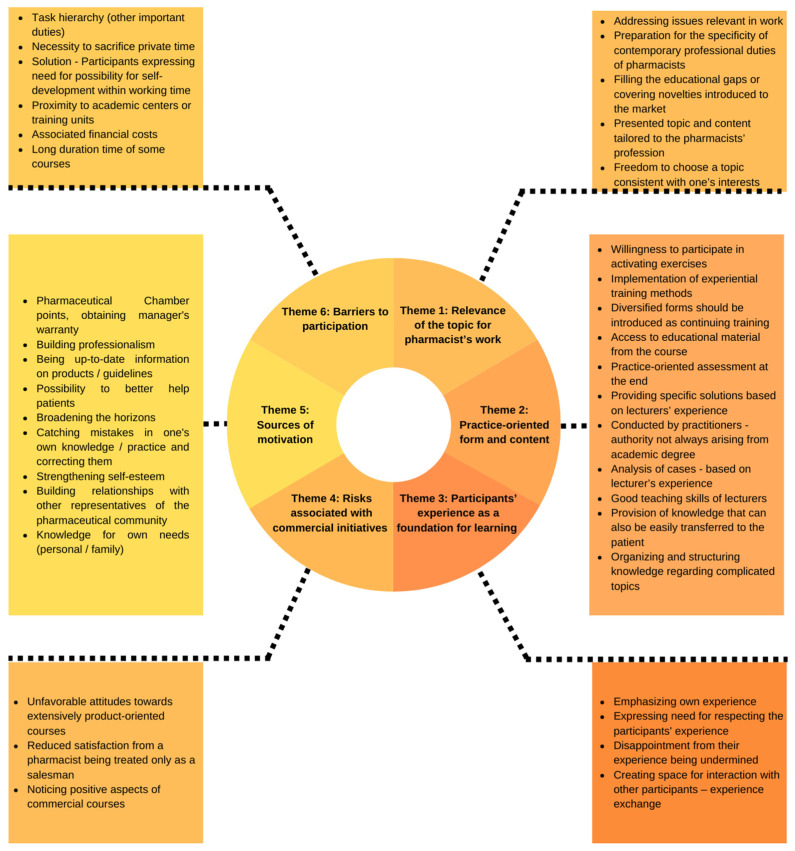
Summary of key findings.

During the conducted interviews, six themes were identified, namely: relevance of the topic for pharmacist’s work; practice-oriented form and content; participants’ experience as a foundation for learning; risks associated with commercial initiatives; sources of motivation; and barriers to participation. Table 3 presents the identified themes with corresponding threads raised by the respondents. The summary of research findings was also presented as Figure 1.

### 3.1. Theme 1: Relevance of the Topic for Pharmacist’s Work

Respondents generally recognized the importance and pharmacists’ interest in participating in lifelong learning initiatives. However, in their decisions to participate in them, they emphasized the vital role of the presented topic and its relevance in their everyday work. For some of them, the sense of the topic’s relevance arises from the prevalence of a given problem, for instance, a disease in the general population or the frequency of patients’ inquiries.

*“I noticed that the most willingly attended [...] are lectures on these basic diseases, namely chronic diseases, diabetes, hypertension, heart disease, asthma. This is what is important to us in our practice in pharmacy.”*

PH5

*“I think it would be very cool to have more dermatology courses. Because there is very little of it during the studies, I think it is not enough, and yet patients come to the pharmacy, showing something and asking what it is.”*

PH8

The relevance of the topic could also be dictated by the specificity of the participants’ place of work and the assortment of products offered, as evidenced by the example of a respondent whose pharmacy has a wide selection of dermatology products.

*“For example, I like courses on cosmetics very much, but this is because I am a woman and our patients are also interested in this subject since we have a well-developed dermo-cosmetics department. Patients ask about it, and without these courses, it would be difficult to systematize this knowledge on your own, to advise them logically somehow.”*

PH6

Furthermore, new drugs, equipment, and other novelties introduced to the pharmaceutical market could also be of interest to pharmacists.

*“I remember well a diabetology course about a new drug entering the market. [...] it was a very, very important training because here in the pharmacy, we dispense a lot of this drug, and I had to train myself in this direction so that I could tell the patient something about it. […] And this course was really helpful for me.”*

PH4

*“We all lack such practical knowledge in the use of medical equipment. There is still not enough of that. It is also an area that is developing. There is new equipment, some new improvements in this equipment, in inhalers, in glucometers. [...] We have little to do with a diabetic patient here, but patients are asking questions.”*

PH9

Last but not least, pharmacists also recognized that relevant courses could prepare them for occurrences that are less frequent but could have serious consequences for the patients.

*“First aid was also such a reminder because you remember most things. But an important reminder, after all, if you do not save a person on a daily basis, then it is really worth repeating. Especially in a pharmacy, it does not often happen that someone here faints, but it can happen, and you have to be prepared for such a situation.”*

PH8

On the other hand, lifelong learning initiatives that pharmacists perceived as having low relevance for their work were not positively assessed by the participants.

*“Unfortunately, the professor conducted the course as if it was for doctors. I didn’t learn much from it. I didn’t understand too much, not even what it was about. He went too deep into details about orthopedics. As pharmacists, we did not necessarily even know what he was talking about [..] it was not strictly for a pharmacist—a typical training for a physician.”*

PH4

### 3.2. Theme 2: Practice-Oriented Form and Content

Apart from the topic of the course, the way it was presented and the educational methods used were also important for the respondents. A more practice-oriented approach was preferred in this context instead of pure lectures. Provided examples of such enhancements included the use of multimedia, demonstrations, or interactive forms such as tests or quizzes.

*“It’s hard for me to say, for sure, it should not be a form of a lecture because, at some point, everything will run away somewhere. More multimedia or demonstration forms, related to seeing something—it is certainly much more interesting than pure listening and sitting at the lecture.”*

PH4

*“Course in the form of slides, but the lecturer had samples/testers of all the products she talked about. We could touch, smell, and smear—this is also very important.”*

PH1

*“First aid—organized by trained paramedics was very interesting, with phantoms, there were a lot of practical classes, it was not only listening or watching slides but actually made you actively participate in these classes.”*

PH9

Another aspect appreciated by the respondents was the materials from the course—useful to refresh knowledge after some time has passed.

*“For me, it is very important to have already communicated at the entrance that for this stage you have an outline, you should use it here, you can make additional notes, the place for notes is also crucial. Because you know, it later runs out of your head, so you can look something out.”*

PH8

Respondents’ practical orientation to learning was also reflected in their descriptions of lecturers and the qualifications or qualities they should possess. Participants seemed to highly value courses given by experienced clinicians, either physicians or clinical pharmacists, who shared their knowledge and real-life practice stories.

*“The lecturer was superbly prepared, and with remarkable ability to conduct the course in a very understandable way, the presentation was concrete without unnecessary details. She also did not use such a very professional language, but the one we use when talking to a patient, understandable to the patient—I also think it is important [...]. She made sure that it was in a form that we can immediately pass on to the patient.”*

PH1

*“I value people who have a lot of knowledge and experience and, in addition to some general knowledge, can point something out, maybe not simply, but specifically—give specific tips. […] I attended several such courses that there was a lecture on the principles of therapy in general, and then a doctor or associate professor said: when a patient comes, do that—he described patterns and procedures. It is great facilitation. It facilitates the work because it clearly shows the proper patient management in the pharmacy.”*

PH3

*“For me, lectures conducted by doctors or clinical pharmacists are more interesting. Those who have practical contact with the patient, not theoretical. […] They talk about anonymous patients, but these are real cases.”*

PH5

The provided accounts of poor-quality courses seem to confirm these observations. They were mainly heavily content-oriented and did not allow for practical application of knowledge or problem-solving.

*“[The worst training] was conducted by this, certainly experienced [sneeringly], professor, it was about herbal medicine. But it didn’t add anything new. It was conducted as I would just read a herbal medicine textbook. It did not bring anything new. I might as well have stayed home and read an old book.”*

PH1

*“Poor-quality courses are when only data on a given topic are presented. Maybe not the subject of the training itself, but the knowledge contained in it did not simply translate into practice. [...] Good online courses are the ones from which a lot can be taken and applied in practice, in our everyday work.”*

PH7

### 3.3. Theme 3: Participants’ Experience as a Foundation for Learning

The prior knowledge and experience seemed to form an important part of respondents’ professional and self-identity. Pharmacists often referred to them, and in this context, lifelong learning courses were seen as opportunities to broaden or refresh this knowledge rather than build it from scratch.

*“If we have dermo-cosmetics, there aren’t many of these courses. I think that there should be more of them because this influences what we can recommend as there are many dermo-cosmetics. Honestly speaking, I know a lot about them, but not as much as I would like to.”*

PH8

*“It often happens that even though I am a pharmacist with many years of experience and the topic seems familiar, sometimes I hear something that I will say to myself: for this one sentence, for this one message, it was worth coming.”*

PH1

Therefore, courses acknowledging, respecting, and utilizing their participants’ prior knowledge and experience as a basis and learning resource were also appreciated by the pharmacists who had occasion to participate in them.

*“Recently, I was at interesting courses, where either preparations were given, and it was necessary to conclude a diagnosis, what it could possibly be, or there was a diagnosis, and it was necessary to choose drugs, and these were also prescription preparations, but if you work for some time, you know these prescription sets, and you can deduce the disease from that.”*

PH5

As this respondent summarized it neatly, the experience exchange, in this case, can work both ways—not only can participants learn from the lecturer but also from each other.

*“If someone has a specific experience, they pass it on, and I add it to my experience. And I can also convey this [my experience] on certain groups of drugs.”*

PH5

In this spirit of a strong belief in their already existing knowledge and competencies, pharmacists were dissatisfied with lecturers who did not appreciate or even undermine their experience.

*“The worst course was also a lecture, but it was really boring—just sitting and listening [...] The subject would be interesting, but the professor approached us as if we were children from kindergarten. Apart from that, not everyone has to know about these herbs well, right? Not everyone has to remember them after these 20 years—that’s why you come to courses to remember some things. But really, professor, we were very, very disappointed with this way of approaching us.”*

PH2

### 3.4. Theme 4: Risks Associated with Commercial Initiatives

Commercial lectures seemed to form a separate category of lifelong learning initiatives that pharmacists participated in. Although some of them valued them for their satisfactory merit, often renowned invited lecturers, and the opportunity to be up to date with new pharmaceutical products, respondents also reported a sense of pressure to sell given product and commercial motivations of the organizers.

*“It was always said [...] that these so-called commercial courses, sponsored, for example, by producers, have such an unappealing subtext that they are organized so that pharmacists recommend drugs from this company [...] But I do not see this as a problem. Of course, the message is that they recommend or present their offer, but then it is up to the pharmacist at work to decide whether they can evaluate this offer and the offer of another manufacturer and what they will recommend to the patient.”*

PH3

*“I poorly rate one [commercial course], but that’s because it was strongly encouraged to sell a given product. You know—[as the lecturer] you can tell [about the product], you can encourage. I am more encouraged if someone tells me something about this product sensibly, then I know that I can recommend it, but not in such a way that I am being forced to sell this product because it is distasteful, non-ethical, and it should not be that way.”*

PH8

This sense of commercial purpose also seemed to affect the way that lecturers presented their topics, thus reducing participants’ satisfaction.

*“Although I really appreciate the professor [the lecturer], sometimes, I don’t know where it comes from, or sponsorship, anyway sometimes he conducts these courses as if the pharmacist was only a seller and should only offer drugs from a specific shelf and specific company.”*

PH5

### 3.5. Theme 5: Sources of Motivation

Respondents provided different sources of motivation for participating in lifelong learning. Admittedly some of them were not surprising. Pharmacists often mentioned the educational points awarded by the Pharmaceutical Chamber, but they did not seem to constitute the main reason for attending lectures and courses. Instead, other sources of motivation were listed, for instance, the urge to acquire new knowledge, qualifications, or being up-to-date with the newest achievements in medicine and pharmacy.

*“I did not verify that [whether the Chamber awards points] […] For me, the knowledge I will acquire there is more important. So not always, or even rarely, I go to the course because it is associated with points, but because it is a requirement—it is a nice addition.”*

PH1

*“This is strictly collecting points, but I still think it should be. Good that it is. It’s good that it is required because the pharmaceutical market and the sciences related to it are constantly developing—we should not be left behind.”*

PH6

The motivation of others arises from the sense of responsibility for patients, their willingness to provide them with the best pharmaceutical care and evidence their professionalism. This all seemed to be perceived as increasing the pharmacists’ authority in patients’ eyes.

*“We can convey this to patients, that we are training, we are more professional, so I believe that this is the main advantage of this training.”*

PH4

*“Courses are self-development in general, for yourself—as a desire to develop your interests. But there are also many benefits for patients, mainly in the process of selecting specific drugs for them [the patients], choosing newer and better ones with fewer side effects.”*

PH5

Personal and family factors were also a source of motivation for some pharmacists. Interestingly, to our best knowledge this is the first account of family factors as motivators for continuous education of pharmacists described in the literature.

*“I am also interested in the latest achievements and new approach to certain drugs […] maybe it is due to my age and my … maybe not ailments, but in my family, for example, I have people with heart and circulatory system diseases who struggle with too high cholesterol. It also applies to me a bit because I think I am genetically burdened. That is why I am interested in the use of these natural preparations or dietary supplements, or the principles of a diet in general that will lower cholesterol.”*

PH3

Finally, lifelong learning initiatives can also be an occasion for social gatherings with friends and colleagues.

*“I am from this older generation of pharmacists, because I have been working for 20 years, more than 25 years, so for me, it fulfills two functions—one—meeting with friends, as we rarely see each other after many years because everyone in their own, by the way, many of my generation’s girls, have their own pharmacies. So one, these social meetings apart from such scientific aspects and two, it forces us to learn.”*

PH2

Some respondents also pointed out that despite the system of points awarded for participation in lifelong learning by the Pharmaceutical Chamber, there are almost no consequences for not fulfilling this duty.

*“There is one more oversight in this continuous training. If we are not the pharmacy managers, there is no consequence if we will not collect that number of points. Only if we wanted to be a manager, we would not get a warranty from the Pharmacy Chamber, and there is no other system of penalties if we will not collect [the points].”*

PH2

### 3.6. Theme 6: Barriers to Participation

As the barrier to participating in lifelong learning initiatives, respondents frequently mentioned the lack of time due to the long working hours and tight schedules. One of their propositions in this regard was to include such courses as a regular part of their job instead of the necessity of sacrificing their free time.

*“They [the courses] are very long. Often, when there are more difficult topics, it takes the whole day, for example, and in fact, it is devoting your own free time. And for me, it is a difficulty. Since the specificity of work is that we work from Monday to Sunday, generating an entire Saturday, for example, is difficult for us. […] If we go to OHS training, which we are obliged to do because of our work, we have it written in a schedule, and we do it as part of our work. Why could not such continuous training be written [in a schedule]? In most pharmacies, it is done in private time.”*

PH6

*“Recently, I participated in the course. It was all Sunday, from the registration at 8:30 a.m. until 7:00 p.m.—it was the whole Sunday, and I was at work the whole week before. I was also at work the previous weekend. Even though I do not participate in it somehow actively, I feel tired.”*

PH9

*“Access to the courses […] because, for example, they very often collide with working hours, so it would be nice if they introduced for it to be within working hours [...].”*

PH10

Other difficulties included getting to and financial costs of some courses.

*“Difficulties, that is, I have a car, so there is no problem with getting there. But some of my friends have a problem if it is outside the city center.”*

PH2

*“Some trivial ones that I can’t, for example, change the schedule at work or financial ones—unfortunately, more interesting courses are also paid, for example, run by specialists.”*

PH5

*“To be honest, I (and I think that most people) choose free courses. There used to be no such choice because they [the courses] were rather paid. At the moment I go where it is free. I think I also have the privilege of living in a large academic center. I don’t have to go anywhere, I don’t have to pay for a hotel, as many of my friends do. [...] Perhaps it would be good to organize such meetings in smaller towns, which would facilitate the participation of people from small towns, from towns further away from academic centers. [...] The Internet here comes under the roof, so it goes to everyone, it is actually a solution, and I also think the future.”*

PH9

Similarly, other respondents also saw online lifelong learning initiatives as an opportunity and a remedy for previously described shortcomings or limitations of traditional lectures or courses. It was especially apparent in the case of their limited time to participate in such initiatives or the need to travel long distances to get to them.

*“[…] But when we have online courses, we can often choose whatever we want. I do not see any obstacle.”*

PH1

*“The form suits me here, especially since I usually do these online courses. Well, let’s face it, this is the most convenient form.”*

PH4

On the other hand, e-learning forms may also be a source of difficulties for some potential participants, for example, those who are more attached and accustomed to direct personal contact.

*“Unfortunately, there are fewer of these courses at the moment, and they mainly refer to these online courses, but for me, direct contact is better. At the moment, I must honestly say that I miss it.”*

PH3

## 4. Discussion

**Figure 2:**
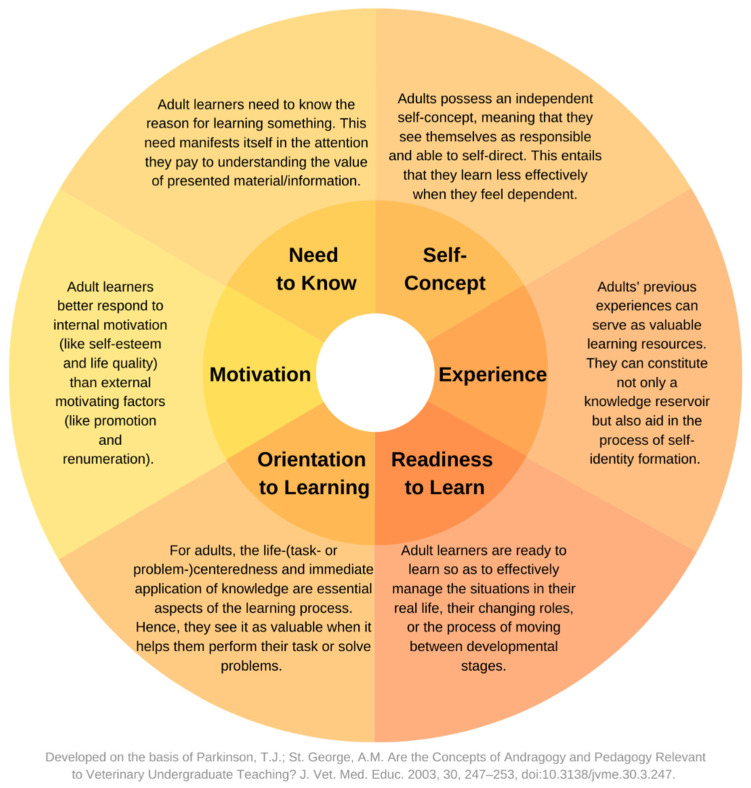
Key assumptions of the andragogy model. Developed on the basis of Parkinson and St. George [29].

Six themes related to pharmacists’ opinions and experiences on continuous education were identified during the study, namely: relevance of the topic for pharmacist’s work; practice-oriented form and content; participants’ experience as a foundation for learning; risks associated with commercial initiatives; sources of motivation; and barriers to participation. We further used our results to draft tips for the formulation of educational interventions within the framework of postgraduate education of pharmacists. During this process we compared and discussed them with previously published literature reports as well as relevant learning theories and presented them below in the Discussion. Among these learning theories we paid special attention to Knowles’ [27] pillars of andragogy, which were identified as “particularly important in professional education” to provide effective and learner-orientated educational solutions [28]. Key assumptions of the andragogy model [29] were presented in Figure 2.

Knowles was criticized that his concepts were not based on sufficient evidence [30]. Although in academic terms, they may be considered obsolete, Thompson and Deis [31] point out that researchers in subsequent years not only did not reject these claims but supported them with additional arguments. Interestingly, a literature search in PubMed with the use of ‘andragogy’ and ‘pharmacy’ as keywords reveals no relevant results on the topic. Thus, the use of well-established educational concepts seems to be still insufficient in the context of pharmaceutical education-oriented research.

Given that *“the degree to which participants react favorably to the learning event”* [14] is an important first step in training evaluation, following them could allow instructional designers to design solutions with a chance to become more effective educational tools.

### 4.1. Tip 1. Consider the Educational Needs and Motivations of Potential Participants

The first step referenced in leading models of designing educational solutions is their consistency with the educational needs of the learner [32,33]. A similar necessity was also observed by Ibrahim [34] in the context of continuing education of pharmacists. Furthermore, individual pharmacies may differ in their specificity, which is largely dependent on, for example, their location. The course offer should, however, consider and respect this specificity. Meanwhile, the studies published so far indicate that even 1/3 of respondents declare a lack of interest in the topics available [35]. To use the potential of the learner’s internal motivation, a diverse range of courses should be offered, allowing learners to choose the one that best suits their needs, so the pharmacist could effectively use the acquired knowledge [36].

Among the benefits resulting from participation in lifelong learning courses, the respondents mentioned both those related to personal development (broadening/refreshing the knowledge possessed, learning about the latest standards, developing one’s interests) and those influencing the quality of their work (professionalism, improvement of patient service, higher self-confidence in contact with the patient). In view of the observed diversification of the motivations of individual respondents, the key to designing continuous education solutions seems to be providing learners with a sense of self-efficacy in designing their own development path. Tjin et al. [36], in a study describing the quality and quantity of pharmacists’ motivation for continuous education, concluded that building autonomy and giving a feeling of mastering certain knowledge and skills can facilitate stimulation of so-called ‘good-quality motivation’—one based mostly on intrinsic aspects.

Meanwhile, online solutions offer unique opportunities in regard to matching the courses with the needs and motivations of potential recipients. Since they are independent of the localization constraints, a pharmacist can freely participate in a course organized in a different city or even country, which can be of particular importance in case of niche topics, for instance. It is also worth emphasizing here that, as Williams et al. [37] indicate, the autonomy-supportive teaching not only contributes to the feeling of bigger competence among learners but also builds in them behaviors that promote patient-centered care. Meanwhile, in the research of Sacre et al. [35], over 30% of respondents reported difficulties finding an adequate program to meet their practice. Moreover, where possible, effort should also be undertaken to enhance pharmacists’ lifelong learning in other, not necessarily formal forms. In fact, informal (e.g., in the workplace or self-directed learning) and formal learning types seem to reinforce each other, not to mention the common acceptance of informal solutions in different continuing professional education systems [38]. One of the pillars of andragogy is self-concept denoting adult learners’ perception of themselves as independent, responsible, and capable of self-direction.

### 4.2. Tip 2. Precisely Define and Communicate the Educational Goals to Potential Recipients

Based on the identified needs, it is crucial to precisely define the qualifications that a person completing the course should have [32]. The goals should not only be communicated to the learner at the beginning of the educational intervention so that the course participant learns more consciously but also to potential learners before the course begins. Thanks to that, the learner could make an informed decision to choose the course best suited to their needs [39]. This may be especially important given the independent self-concept of adult learners and their need for making their own decisions on their education [27,29]. Presenting precise learning objectives of each course would also attract people interested in a given topic, making it easier for them to engage in acquiring new knowledge. Moreover, according to the self-determination theory (SDT) [40], autonomy is one of the factors that must be met in order to inspire the learner’s internal motivation to learn.

### 4.3. Tip 3. Adapt the Content and Educational Methods to Previously Defined Learning Outcomes

When designing each stage of the course, the previously defined learning outcomes should be remembered to avoid participants’ disappointment resulting from spending their precious and naturally limited time participating in the course that did not fulfill the original promise. It should be noted that in line with the constructive alignment principle, *“the key is that all components in the teaching system—the curriculum and its intended outcomes, the teaching methods used, the assessment tasks—are aligned to each other”* [41]. Therefore, the way the course’s goals are formulated should be carefully thought through. It should be remembered that all subsequent decisions regarding, for example, the content or the educational methods used will be secondary to these defined learning outcomes [42]. After all, the given course will be designed differently if its learning outcome is to be, for example, ‘the participant characterizes the side effects of drug X’, and differently if it is ‘the participant detects side effects caused by the drug X’. When formulating learning outcomes, subject-centeredness should be changed to performance-centeredness. The respondents in this study expect that participation in courses will give them more confidence in working with patients, thus confirming the previously described relationship between the sense of their own qualifications and the readiness to perform professional tasks by pharmacists in community pharmacies [43]. Although this tip may seem more specific for academic courses than continuous education, constructive alignment is also relevant in postgraduate education. For example, we believe it is connected with the ‘need to know’ and ‘orientation to learning’ pillars of andragogy. Other researchers also pointed out its value in postgraduate education [44].

### 4.4. Tip 4. Keep the Course Practice-Oriented and Avoid Content-Overload

The respondents in our study indicated that sometimes the content presented during the courses was inadequate to the specificity of their work. This seems coherent with the ‘orientation to learning’ and ‘readiness to learn’ pillars of andragogy [27,29]. Adult learners prefer problem-centerdness over content-centerdness, immediate application of knowledge, and topics relevant to their real life. In the study by Poudel et al. [45], for as many as 30% of respondents, poor quality and methods of educational solution delivery were limiting their participation in continuous education. Therefore, the content selected should neither be too detailed nor too general. It should relate as much as possible to the scope of professional tasks entrusted to the course recipients and enable them to achieve the intended learning outcomes. Our respondents also seemed to prefer more practice- rather than a content-oriented approaches, complaining about content overload and the low perceived value of traditional lectures. Although still frequently used, such passive formats tend to create a disproportion between an extensive amount of provided content and participants’ ability to focus and acquire knowledge, with an attention span of approximately only 10–20 min. For that reason, other forms should be sought in order to minimize the cognitive activity, for example, dividing the content into smaller parts, called “chunking,” can improve focus, learning, and retention of knowledge [46].

Due to the problem-centered attitude of adult learners, the presented content should be as practical as possible. For instance, instead of listing drug interactions on the slide, the learner could be redirected to the interaction checker website (e.g., https://www.drugs.com/drug_interactions.html accessed on 4 May 2022) to assess the interactions on their own. In this way, the role of the trainer evolves, and the teacher ceases to be only a knowledge provider and becomes a moderator of the learning process [47]. By analogy, instead of giving the learners a fish, they are taught to fish—they are familiarized with tools, methods, and algorithms and taught how to use them. Thanks to this, they will be better prepared to react to unique situations and build their self-confidence in solving professional problems. Application of problem-based learning to a course can, for instance, take the form of scenario-based learning or case studies. The use of scenario-based learning means involving the learner more actively by presenting them with hypothetical situations arising from the real-life practice. It gives them the opportunity to apply their knowledge, develop and practice skills, and use their critical thinking, problem-solving, and decision-making skills in the context of real professional life [48,49]. Meanwhile, according to the situated learning theory, it is in the context of its future use that the learning process takes place the best [49]. The research conducted so far also confirms the high interest of pharmacists in distance learning techniques such as virtual patients, for instance [50].

### 4.5. Tip 5. Respect and Utilize Learners’ Experience

One of the crucial elements that distinguish the andragogical approach from pedagogy is the experience of learners [27,29]. Adult learners come with their own baggage of previous knowledge and experiences. This can be particularly important in postgraduate education, where these experiences may arise both from formal education during the studies and subsequent professional experience. As our study shows, in line with the andragogy assumptions presented above, when this experience is appropriately acknowledged it may serve as an important asset of the course and a valuable learning reservoir. Conversely, when it is ignored, it may leave the participants dissatisfied and discouraged. It is worth emphasizing that some studies [51] show that more experienced pharmacists tend to present more positive attitudes toward participation in continuous education than their younger colleagues. Several of our respondents emphasized their existing experience—one even directly verbalized her disappointment from being treated as *“kindergarten children.”* A good strategy is to give the participants space for self-reflection, activating prior knowledge and relating the acquired knowledge to their previous experiences. Given that new information integrates better with the existing one, such activation of prior knowledge may prove beneficial for the learning process and information recall [52]. For instance, case-based learning may be used to achieve that with the lecturer rather as a facilitator than a teacher, guiding the participants by asking questions and stimulating discussion, thus exploring the existing knowledge and identifying gaps [53].

Additionally, both in this and previous studies [45,51,54,55], the pharmacists’ lack of time was indicated as a significant barrier to undertaking additional educational activities. Moreover, in the study by Aldosari et al. [51], over 1/3 of the respondents indicated irregularity in the organization of educational interventions as a barrier. Meanwhile, modern technologies (e.g., the use of authoring tools) allow, to a large extent, to assess and respect the experience of the participants and individualize the learning process. From the learners’ perspective, one of the key values of asynchronous e-learning is the possibility of learning in the time and place most convenient for them [56]. If, as suggested in the previous tip, the designed course consists of small, easily digestible portions, it will allow to better adjust its realization time to the individual abilities of the learner [39,57]. Additionally, if these portions are properly titled and arranged logically, it will make it easier for pharmacists to return to a given topic whenever required.

It also seems reasonable to design a pre-test before the course, the results of which would allow experienced participants to skip selected areas of the course that include already possessed qualifications. Meanwhile, others, who do not have them, would have to pass these parts of the course. The use of the authoring tool to individualize the educational path of the participant also gives the creators of the course the opportunity to provide the learner with immediate feedback on test results. In such feedback, it is worth indicating parts of the course that the learner should pay special attention to and those that may possibly be omitted.

### 4.6. Tip 6. Choose the Lecturers Who Will Be an Authority for the Learners

According to Sato et al. [58], *“experts can teach based upon contextualized thinking, while the novices teach regardless of specific content, cognition, and context. […] this is the reason why experts can respond quickly and intuitively to events and creatively improve their teaching. This is also the reason why novices cannot be flexible in their teaching*.” In fact, our respondents paid much attention to the lecturers of the attended courses, and their expectations were twofold—they should be experts in the field (e.g., experienced clinicians, physicians, or clinical pharmacists) and good didactics offering a variety of instructional methods—practice-oriented, attractive, and interactive. Meeting only one of these requirements was deemed as unsatisfactory, as evidenced, for example, by the above-cited comment of PH1 on the herbal medicine course. Therefore, in the process of lecturer selection, it seems extremely important not only to choose the leading experts in the field but also to pay attention to their didactic skills or offer them some more support in preparing the classes. Such faculty development initiatives to improve teaching effectiveness can take numerous forms, including workshops, fellowships, scholars’ programs, longitudinal programs, short courses, seminar series, peer observations and coaching, self-study audiotapes and CD-ROMS, or web-based training modules, for instance [59]. Moreover, as evidenced by our previous experiences, even relatively inexpensive and simple forms can be perceived as resourceful and helpful by the participating teachers [60].

It should also be emphasized that a given course does not have to be entirely conducted by one person. In this aspect, especially the asynchronous e-learning model allows for delivering solutions prepared by a diverse team of specialists. Subject matter experts should also pay attention to the resources they use. The respondents emphasized that they perceive the courses as updating the knowledge they already have. Hence, the literature on which the courses are based should include the current guidelines and the latest studies because, as Knowles points out, the value of the presented materials is subject to the special attention of adult learners.

### 4.7. Tip 7. Ensure a Safe Space for the Interactions between Participants

According to SDT [40], autonomy is one of the factors that must be met to inspire the learner’s internal motivation to learn. The need for relatedness must also be satisfied in this aspect, which can be achieved by treating students with warmth and respect [35]. Such a combination of internal motivation and safe space could, in turn, increase participants’ willingness to interact with each other. Meanwhile, the importance of the social aspect is evidenced in this and other studies. For example, for Flemish community pharmacists keeping in touch with colleagues was one of the factors influencing the motivation [54]. Hence, the course should create an atmosphere of inclusiveness and encourage participants to communicate and discuss [53].

Creating a safe training space in which participants can freely engage in the learning process is also crucial from the perspective of the Social Learning Theory [61]. An interactive environment is essential to conclude that social learning takes place. The sense of security translates into an increased self-effectiveness related to engaging in the presented content. This aspect should also be considered when creating e-learning classes. Digital relationships are detached from social anxiety, which translates into higher self-efficacy in undertaking learning [62,63].

It should be kept in mind that e-learning, despite its many advantages, also has its limitations and may cause some difficulties for instructional designers, for example, in building interactions between course participants. In such a case, to create a space for the exchange of experiences and opinions, it is worth setting up a set of quick contact paths with the use of, for instance, social media or built-in solutions in e-learning platforms (e.g., a forum) [64]. Participants should be given a chance to implement the solutions in practice and encouraged to share their thoughts with the facilitators and other participants.

### 4.8. Tip 8. Provide Participants with Reliable Educational Materials

Both in this study and the ones described in the literature, pharmacists expect to be able to use the acquired knowledge in working with the patient. In a study by Driesen et al. [54], gathering practical knowledge to improve information provision skills was a strong motivating factor for 91% of participants. For example, if the topic of the course is a specific drug substance, it should clearly specify what information about this drug should be provided to the patient. Pharmacists also seem to expect that, as part of the course, they will gain access to high-quality materials presented in an understandable way [45]. If its learning outcomes are oriented toward a specific scope of patient education, educational materials in the .pdf format could be provided, allowing the pharmacist to print them and give them to the patients. One of the motivations to participate in the course may be the willingness to build authority in the eyes of the patient and the image of pharmacists as highly qualified personnel. Therefore, elements of the course facilitating these motivations could be an added value to the offered educational solution for a pharmacist.

### 4.9. Tip 9. Avoid Extensive Product Promotion

Lifelong learning solutions’ participants want to see them as credible and merit-based. In our study, respondents noticed shortcomings of commercial lectures in this aspect, sensing commercial motivations of the organizers and subconscious pressure to offer the promoted product to patients in the future. It should be mentioned here that there are currently no limitations on the organization or financing of such events by the companies in Poland. However, as our study shows, their organizers should pay special attention to ensure their scientific merit and integrity. As Knowles points out, adult learners want to be independent [27,29]. Therefore, such a sense of external pressure seemed to reduce their satisfaction with a given course. Moreover, as evidenced in this study, some pharmacists can also view it as reducing their professional role to mere sellers of drugs. Meanwhile, the awareness and self-concept of pharmacists as a professional group nowadays seem to be getting stronger. A qualitative study conducted by Salim and Elgizoli [65] on Sudanese community pharmacists in total identified nine additional identities, including, for instance, that of the drug expert, patient counselor, or health promoter. Their study also showed pharmacists’ anticipation of expanding their professional roles and demand for continuous education. Recent studies on Polish pharmacists also revealed their expectations and potential to expand their professional roles beyond that of drug sellers in the context of interprofessional collaboration with physicians [66,67]. Therefore, in line with meeting the expectations and needs of recipients, offered courses should reflect and respect this need.

### 4.10. Tip 10. Take Steps to Evaluate the Course on Different Levels

It should be remembered that the pharmacist participated in the course or educational intervention for some specific reason. Therefore, efforts should be made to assess whether they met the expectations and produced desired outcomes. At this point, it is also worth encouraging the learner to self-reflect. Depending on the situation, opportunity, and context, such evaluation can take place on different levels and dimensions of the aforementioned revised Kirkpatrick Model [14], namely Level 1: Reaction (Satisfaction, Engagement, Relevance), Level 2: Learning (Knowledge, Skill, Attitude, Confidence, Commitment), Level 3: Behavior (Critical Behaviors, Required Drivers, On-the-Job Learning), and Level 4: Results (Leading Indicators). For example, participants could also be asked to retake the test described in Tip 4. The last slide of the course could contain a comparison of learners’ answers before and after the course—this could give the learner a sense of effectively spent time. Assessing participants’ self-efficacy in using the acquired qualifications in practice would also be a valuable addition. According to Bandura, self-efficacy seems to constitute a link between knowing what to do and doing, allowing for an active pro-doing approach when it is high but also resulting in avoidance and fear in case of low self-efficacy [68,69]. The respondents in our study repeatedly emphasized how important it is for them to acquire qualifications that they will be able to use in contact with the patient. However, as evidenced above, in addition to the qualifications held, taking a specific action also requires presenting a specific level of self-efficacy. Meanwhile, studies conducted so far showed positive effects of continuing professional development and education initiatives on pharmacists’ self-efficacy in the context of patients experiencing intimate partner violence [70] or counseling on smoking cessation [71], for example.

In addition, in the case of online courses, it is worth mentioning that the current possibilities of LMS (Learning Management System) platforms also provide the opportunity to verify learners’ involvement and work patterns in the course. The analysis of these statistics could become a self-reflection opportunity for the teacher and help to improve the created educational solution.

We acknowledge the limitations of this study. Firstly, convenience sampling was used, which may suggest the limited scope of the study. It is worth emphasizing, however, that in the absence of existing data in the field of community pharmacists’ lifelong learning, the aim of the study was to understand the point of view of the respondents and embed it in an adequate learning theory context. During the study, we were more focused on obtaining a detailed comprehension of personal insights, opinions, and past experiences of our participants than making generalizations on the national level. This was reflected in our choice of phenomenology as the qualitative approach in the study. Consequently, our results are not meant to be viewed as representative for the wider population. Instead, they are very individual and subjective insights on participants’ way of thinking about continuous education. Additionally, diversification of the participants in regard to factors such as their gender, age, professional experience, or specialization, allowed to broaden the research perspective by capturing different opinions and perspectives potentially present in the pharmacists’ community. We believe that despite the local context of the study, it can provide valuable information on pharmacists’ perspectives towards continuous education. Moreover, by putting them together with relevant learning theories, it can be used by the international audience as a helpful tool in developing lifelong learning solutions. Demonstrating the connections between pharmacists’ preferences in the field of lifelong learning and theories described in the literature may also inspire researchers to further quantitative exploration of the topic based on learning theories. Another limitation is that all respondents declared their commitment to participate in continuous education, which may result from the fact that the study recruitment process could have attracted individuals with a more favorable attitude towards lifelong learning. Therefore, it would be worthwhile to conduct further research based on judgmental sampling and invite those who do not participate in such continuous education courses to participate in the research.

## 5. Conclusions

**Figure 3:**
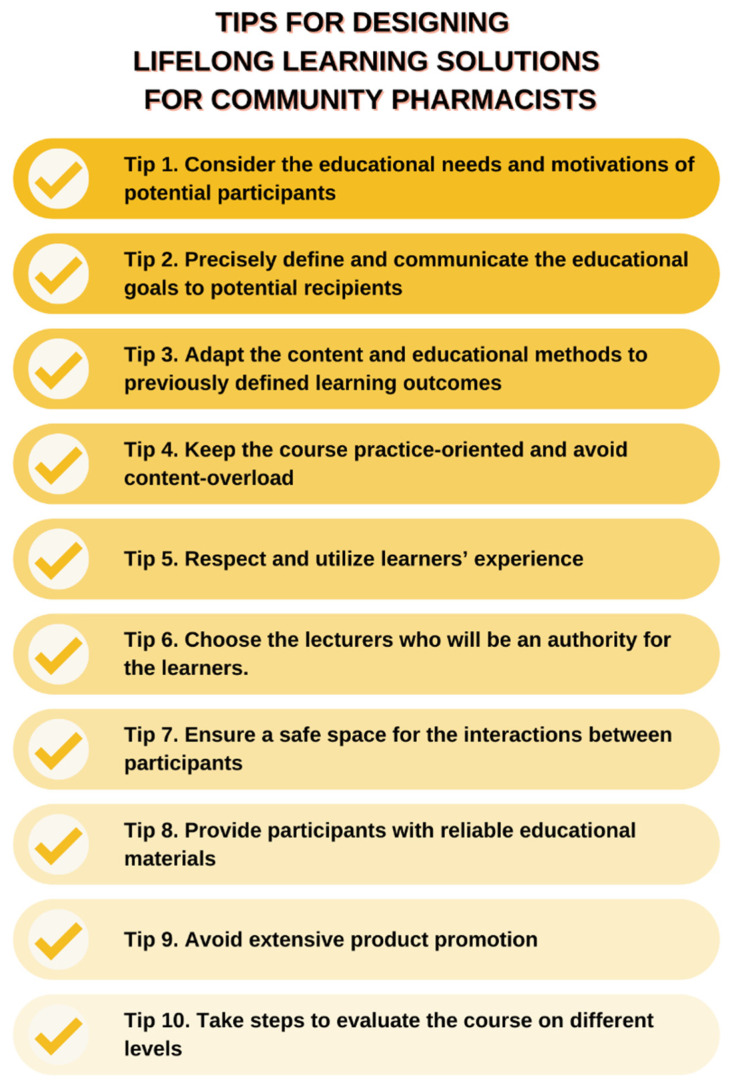
Ten tips for designing lifelong learning solutions for community pharmacists.

Based on the expectations of pharmacists and the conducted literature review, ten tips were developed for designing more effective lifelong learning solutions for community pharmacists according to learning theories with a special emphasis on pillars of andragogy, taking into account both the organization of the educational process and design of single courses (Figure 3). Due to the growing popularity of online education, the proposed tips attempted to cover both traditional face-to-face and remote online solutions. Moreover, given the limitations of the online environment, educational solutions designed in this way should consider the use of a combination of asynchronous and synchronous approaches to, on the one hand, allow pharmacists to gain knowledge at a convenient time, and on the other hand, allow the personal contact with an expert and limit users’ loneliness.

## Figures and Tables

**Table 1:** Characteristic of the respondents’ demographics.

Participants’ Code	Age Range [years]	Gender	Specialization
PH1	50–60	female	no
PH2	60–70	female	no
PH3	50–60	female	yes
PH4	20–30	female	no
PH5	50–60	female	yes
PH6	20–30	female	no
PH7	30–40	male	no
PH8	20–30	female	no
PH9	50–60	female	no
PH10	20–30	female	no

**Table 2:** The interview guide used during the study.

Topics with Questions Covered by the Interview Guide
1. Opinion on the need for continuous training of pharmacists (opening question) - Under the law, pharmacists are required to participate in lifelong learning. What is your opinion on this subject? - What are the first associations that come to your mind? 2. Benefits of undertaking lifelong learning - What are the benefits of involving a pharmacist in lifelong learning? - What can be the worth of the participation of pharmacists in lifelong learning? - What may encourage pharmacists to participate in it? 3. Barriers to undertaking lifelong learning - What may discourage pharmacists from participating in lifelong learning? - Which factors may limit pharmacists’ access to lifelong learning? 4. Previous experience with participation in lifelong learning - Please think about the course you remember the most, the best and the worst course you have participated in. Can you tell me more about them? 5. Expectations for lifelong learning - How, in your opinion, should the continuous education of pharmacists in Poland be organized? - Please try to imagine the perfect course/lifelong learning initiative—one that could be of value to you. What should it look like? 6. Individual possibilities of undertaking lifelong learning - How do you assess the availability of lifelong learning initiatives? - How do you assess the involvement of pharmacists in lifelong learning? 7. Potential additional observations (closing question) - Do you have any reflections on lifelong learning that I have not asked about so far and would like to share them? - I would like to learn about your other thoughts on lifelong learning, which, in your opinion, are important and worth attention.

**Table 3:** Themes identified during the study with relevant threads raised by the respondents.

Theme	Threads Raised by Respondents	Number of Respondents
THEME 1: Relevance of the topic for pharmacist’s work	Addressing issues relevant in work	8
	Preparation for the specificity of contemporary professional duties of pharmacists	5
	Filling the educational gaps or covering novelties introduced to the market	4
	Presented topic and content tailored to the pharmacists’ profession	3
	Freedom to choose a topic consistent with one’s interests	2
THEME 2: Practice-oriented form and content	Willingness to participate in activating exercises	6
	Implementation of experiential training methods	5
	Diversified forms should be introduced as continuing training	3
	Access to educational material from the course	1
	Practice-oriented assessment at the end	1
	Providing specific solutions based on lecturers’ experience	7
	Conducted by practitioners—authority not always arising from academic degree	5
	Analysis of cases—based on lecturer’s experience	3
	Good teaching skills of lecturers	5
	Provision of knowledge that can also be easily transferred to the patient	7
	Organizing and structuring knowledge regarding complicated topics	4
THEME 3: Participants’ experience as a foundation for learning	Emphasizing own experience	4
	Expressing need for respecting the participants’ experience	2
	Disappointment from their experience being undermined	1
	Creating space for interaction with other participants’experience exchange	3
THEME 4: Risks associated with commercial initiatives	Unfavorable attitudes towards extensively product-oriented courses	3
	Reduced satisfaction from a pharmacist being treated only as a salesman	1
	Noticing positive aspects of commercial courses	3
THEME 5: Sources of motivation	Pharmaceutical Chamber points, obtaining manager’s warranty	4
	Building professionalism	3
	Being up-to-date information on products/guidelines	5
	Possibility to better help patients	6
	Broadening the horizons	4
	Catching mistakes in one’s own knowledge/practice and correcting them	2
	Strengthening self-esteem	3
	Building relationships with other representatives of the pharmaceutical community	1
	Knowledge for own needs (personal/family)	5
THEME 6: Barriers to participation	Task hierarchy (other important duties)	4
	Necessity to sacrifice private time	6
	Solution’Participants expressing need for possibility for self-development within working time	4
	Proximity to academic centers or training units	3
	Associated financial costs	4
	Long duration time of some courses	5

## Data Availability

The data used to support the findings in this study are available from the corresponding author upon request.

## Supplementary Materials

The following supporting information can be downloaded at: https://www.mdpi.com/article/10.3390/healthcare10071167/s1, File S1: Excerpts from the interview with PH2 along with the researchers’ notes.
