# Supplementary material for: Learning Theory-Driven Tips for Designing Effective Learning Solutions for the Continuous Education of Community Pharmacists to Enhance Patient-Centered Care—A Qualitative Study
Source: Healthcare (Basel). 2022 Jun 22;10(7):1167. doi: 10.3390/healthcare10071167 (PMC9320098; doi:10.3390/healthcare10071167)
Supplement: Supplementary file 1 [file healthcare-10-01167-s001.zip › healthcare-1735205-supplementary.pdf]

Excerpts from the interview with PH2 along with the researchers' notes

Transcripts	Exploratory comments (researcher 1)	Exploratory comments (researcher 2)	Emerging themes
**Researcher: We as pharmacists, should participate in lifelong learning. What do you think about it?**			
I am from this older generation of pharmacists, because I have been working for 20 years, more than 25 years, so for me, it fulfills two functions – one - meeting with friends, as we rarely see each other after many years because everyone in their own, by the way, many of my generation's girls, have their own pharmacies. So one, these social meetings apart from such scientific aspects and two, it forces us to learn.	She highlights her experience<br>Smiling. Recalling meetings with friends evokes positive emotions. "Many girls" - she feels younger among her peers.<br>Laughter. It's awkward to admit it's not just rising qualifications - aware of different social expectations.	Emphasis on professional experience<br>Courses as occasions for social meetings – additional benefit<br>Learning on second place – less important???	Adult persons' experience - andragogy (experience)<br>Social dimension of participation in courses. The need to interact with others - social learning
At least for me, I have to come home and verify some of my knowledge. It is also a good thing for me because I would not have such a whip over myself...	Participation in the courses motivates to further self-education after training hours.<br>Disappointed that the system does not enforce greater commitment to raising qualifications.	Courses as motivating factors	
There is one more oversight in this continuous training. If we are not the pharmacy managers, there is no consequence if we will not collect that number of points. Only if we wanted to be a manager, we would not get a warranty from the Pharmacy Chamber, and there is no other system of penalties if we will not collect it [the points].	She expects a system of rewards and penalties	Mostly no consequences for not participating – disappointment<br>Carrot and stick as desired model???<br>"System of penalties"	The value of external motivation<br>Expectations for system solutions
Apart from the fact that I just like it, I am one of those who like to go to these courses, like to learn various things there, so I mean, it's cool for me. However, I think it would mobilize me more if I had like a whip over my head.	She likes to learn. She likes going to courses.<br>Internal motivation seen as insufficient to take action - need for external motivation	Positive attitudes<br>Nevertheless, need for external motivation. Kind of paradox???	Internal motivation<br>Internal need to have external motivation

Transcripts	Exploratory comments (researcher 1)	Exploratory comments (researcher 2)	Emerging themes
**Researcher: If you could describe a training that you remember the most?**			
The worst course was also a lecture, but it was really boring - just sitting and listening.	She is disturbed by passivity in training - example not influenced by content of the course.	"just sitting and listening" – dislike for passive learning	Practical orientation to learning – andragogy
By the way, the professor works at the University [here name of the university]. Anyway, it was a training conducted by the Pharmaceutical Chamber - not so long ago, maybe 2 years ago.	She draws attention to the position / authority of the teacher<br>She points out that the training was branded by the Pharmaceutical Chamber	Authority of those responsible for the course - just a mention (by the way) or seen as potential guarantees of courses' merit??? Does it enhance the disappointment?	Facilitators - the authority of the lecturer<br>Facilitators - training branded by the Chamber
**Researcher: Do you remember the subject of the course?**			
The subject would be interesting, but the professor approached us as if we were children from kindergarten. Apart from that, not everyone has to know about these herbs well, right? Not everyone has to remember them after these 20 years - that's why you come to courses to remember some things. But really, professor, we were very, very disappointed with this way of approaching us.	She is disappointed / offended by the way she was treated by the teacher.<br>She expects space for not knowing sth / making mistakes?<br>She expects respect due to her long professional experience.<br>She is not satisfied with communicating only dry facts – she appreciates course'spracticality.	"kindergarten children" !!!<br>role of acknowledging participants' experience / treating as adults<br>need for a "safe space" – mutual respect<br>"that's why you come to courses" !!!<br>Preferring practical approach over theory	The need to appreciate her experience and respect the length of work - andragogy
**Researcher: If you could imagine an ideal course, in which you would like to take part? How should it look like?**			
Yyy... not just talking... Among such interesting courses it was probably [the name of the training brand]. It was combined with practical training in first aid. First aid and we could try artificial respiration on phantoms. Even during the break, those who did not get to this training, so to speak, we could touch or even use a defibrillator, so it was in such a really interesting form. It is sometimes said that pharmacists are not allowed to give first aid. And here we were told "there is no that you are not allowed - you can - and even you must" - because this is the ministry's ordinance that we will be punished if we do not provide first aid - if someone knows how to do	Some participants took the extra time to practice first aid in practice.<br>The opportunity to practice is of great value - the phrase "they did not get to"<br>She wants to be able to get involved in helping, but is not sure how much she is allowed to do. The training gave her permission to help the patient. It provided her with legal basis giving her "legitimization" to help. Doing sth practically raises the intention to help	Sacrificing the break signifies the importance of the topic/course.<br>Afraid to help despite wanting to.<br>Benefits for potential patients in need – now she knows she can/must help, knows how.<br>Preferring practical approach over	Expectation of association with the professional practice - andragogy (orientation to learning)<br>Importance of the topic.<br>Internal Motivation

it, or has undergone any training. But on the other hand, it has always been said that maybe better not because of some penal consequences, if you do something to this man, if you help him wrong, they will take you to court. And here, you can practically dare yourself to try, right?

theory.	
	Rising knowledge, skills, awareness.
Occasion to practice in "safe" conditions	Building self-esteem
